# Rheoencephalography: A non-invasive method for neuromonitoring

**DOI:** 10.2478/joeb-2024-0003

**Published:** 2024-03-13

**Authors:** Sandor Szabo, Zsolt Totka, Jozsef Nagy-Bozsoky, Istvan Pinter, Mihaly Bagany, Michael Bodo

**Affiliations:** 1 University of Szeged, Faculty of General Medicine, Department of Aviation and Space Medicine. Kecskemet, Hungary; Hungarian Defence Forces Medical Center, Aeromedical, Military Medical Screening and Healthcare Instituter; Kecskemet, Hungary; 2 John von Neumann University, Kecskemet, Hungary; 3 Uniformed Services University of the Health Sciences, Bethesda, MD, USA

**Keywords:** Intracranial pressure, pulse wave morphology, rheoencephalography, cerebral blood flow, autoregulation, breath-holding, head-down-tilt position, noninvasive

## Abstract

In neurocritical care, the gold standard method is intracranial pressure (ICP) monitoring for the patient's lifesaving. Since it is an invasive method, it is desirable to use an alternative, noninvasive technique. The computerized real-time invasive cerebral blood flow (CBF) autoregulation (AR) monitoring calculates the status of CBF AR, called the pressure reactivity index (PRx). Studies documented that the electrical impedance of the head (Rheoencephalography – REG) can detect the status of CBF AR (REGx) and ICP noninvasively. We aimed to test REG to reflect ICP and CBF AR.

For nineteen healthy subjects we recorded bipolar bifrontal and bitemporal REG derivations and arm bioimpedance pulses with a 200 Hz sampling rate. The challenges were a 30-second breath-holding and head-down-tilt (HDT – Trendelenburg) position. Data were stored and processed offline. REG pulse wave morphology and REGx were calculated.

The most relevant finding was the significant morphological change of the REG pulse waveform (2^nd^ peak increase) during the HDT position. Breath-holding caused REG amplitude increase, but it was not significant. REGx in male and female group averages have similar trends during HDT by indicating the active status of CBF AR.

The morphological change of REG pulse wave during HDT position was identical to ICP waveform change during increased ICP, reflecting decreased intracranial compliance. A correlation study between ICP and REG was initiated in neurocritical care patients. The noninvasive REG monitoring would also be useful in space research as well as in military medicine during the transport of wounded service members as well as for fighter pilots to indicate the loss of CBF and consciousness.

## Introduction

### Intracranial pressure

ICP is one of the most important methods in neurocritical care. Doctors need to determine the numeric value and changes of ICP, whether before or after an operation [1–10]. In recent years, the ICP waveform analysis has got more attention [10–20]. Normal ICP waveforms have 3 peaks: P1 = percussion wave, which represents arterial pulsation; P2 = tidal wave, which represents intracranial compliance (ICC); and P3 = dicrotic wave, which represents aortic valve closure and venous pulsations. In a normal waveform, P1 is the highest peak resulting in a “P1>P2” or “good ICC” morphology. In the case of a “poor ICC waveform”, P2 is higher than P1 (P2>P1), indicating compromised ICC. An additional worsening of ICC results in a “triangle shape waveform” where P2>P1 and P3 result in the 3 waves fusing into a triangular shape. This publication uses REG peak labeling identical to ICP pulse waves. When the tidal peak (P2) acquires an amplitude higher than the upstroke peak (P1) it is known that the intracranial space has lost its compensatory reserve, with impairment in ICC. The problem with ICP measurement is that it is invasive. The research goals are to substitute with noninvasive methods.

### Cerebral blood flow autoregulation

CBF AR is the mechanism that safeguards blood flow to the brain in the context of changes in arterial blood pressure (ABP), the most important determinant of CBF. AR operates by regulating cerebrovascular resistance to counteract changes in blood pressure [21,22]. About acute adjustments, these changes are thought to mainly occur due to actions of pressure-sensitive vascular muscle that alter vascular resistance through vasodilation or vasoconstriction. Brain function critically depends on closely matching metabolic demands, appropriate delivery of oxygen and nutrients, and removal of cellular waste. This matching requires continuous regulation of CBF, which can be categorized into four broad topics:

1. autoregulation, which describes the response of the cerebrovascular to changes in perfusion pressure;
2. vascular reactivity to vasoactive stimuli including CO_2_;
3. neurovascular coupling, i.e., the CBF response to local changes in neural activity (often standardized cognitive stimuli in humans); and
4. endothelium-dependent responses [18].

Despite continuous technological advances that provide more detailed insight, brain imaging techniques are still limited by a poor temporal resolution that would be needed to record rapid changes in CBF in response to physiological or pathophysiological stimuli [23–26]. Computerized, real-time invasive CBF AR monitoring is possible using a program to calculate pressure reactivity index - PRx [27]. The fundamental relationships between ABP, vessel tone, cerebral blood volume, and ICP form the PRx basis. PRx is analogous to other time-domain AR indices and is calculated as the continuous correlation between 30 consecutive time-averaged (10 s) ABP and ICP values. A positive index (positive correlation) implies impaired CBF AR, while a negative index (inverse correlation) implies intact AR [27,28]. It was demonstrated that PRx can be calculated from REG, called REGx [29].

### REG

The USA FDA acknowledged REG and the method of measuring CBF [30]. An in vitro study demonstrated that the electrical bioimpedance method (REG) can reflect volume change [31]. It was published that REG reflects ICP elevation [32,33]. REG influencing factors were summarized by Moskalenko [34]. A human study demonstrated that REG reflects cerebral volume increase during CO_2_ inhalation, like ICP [32]. Early publications stated that REG reflects ICP and cerebrovascular vascular disease [35,36]. Since REG is an electrical impedance measurement, its units are in ohm. The bipolar method uses two electrodes. The pulsatile component of REG is a small portion of the total impedance. Using digital data processing the REG pulse wave numbers are either in Volt or units of analog-digital conversion. In our data processing, we measured the peak amplitudes in control and test conditions, expressing the latest values in the percentage of control. In such cases, the original ohm units have no meaning.

Several REG correlation/validation studies were published [37–44]. A study demonstrated that REGx is identical to PRx [29]. The clinical importance of this fact is that REG is non-invasive in humans. It was published that ICP pulse wave distortion during ICP elevation indicates decreased intracranial compliance [18–20]. A recent publication proved that REGx reflects decreased IC and REG pulse morphology change is identical to ICP morphology alteration during decreased IC compliance, i.e. P2 is higher during passive CBF AR [45]. Several CBF AR tests were compared in a human study showing that CBF reactivity was best reflected by REG and NIRS by using a 30-sec breath-holding [41]. Based on Jenkner’s data [36] healthy vs. arteriosclerotic REG pulse wave anacrotic/rising time, the threshold was set at 180 msec [46,47]. Jenkner also described the increased third peak amplitude of the REG pulse wave during the HDT position [36]. REG was used in aviation and space research [48–50]. Based on these, REG is a potential noninvasive surrogate of invasive ICP.

### Space research aspects

CBF and its AR are important in military aviation and space medicine. Impaired CBF AR and reduced CO_2_ reactivity after long-duration spaceflight were described [51]. The cerebral vasculature is sensitive to changes in both the arterial carbon dioxide (CO_2_) and oxygen partial pressures so that hypercapnia/hypoxia increases and hypocapnia/hyperoxia reduces global CBF. For details about the central CO_2_ chemoreception neural mechanism and CBF response, see [52,53]. To imitate conditions on the International Space Station (ISS), a ground-based model of microgravity studies used the head-down tilt (HDT) or Trendelenburg position [54–56]. The increased CO_2_ concentration on ISS causes increased brain blood volume, an additional factor in increased ICP. NB: In neurocritical care practice, patients’ heads in bed are elevated to decrease ICP.

### Spaceflight Associated Neuro-ocular Syndrome

In space research, one unsolved problem is Spaceflight Associated with Neuro-ocular Syndrome (SANS) [57,58]. It is a secondary risk and relevant gap (Solicitation Number: 80JSC019L0001; 2019). SANS is a unique condition with no perfect terrestrial analog. The recent list of SANS gaps (n=14) doesn’t mention CBF or its AR. Also, it was out of focus that the ophthalmic artery is a branch of the internal carotid artery. The cerebrovascular aspect of SANS has not been investigated previously. It was suspected that ICP is a contributing factor, but ICP was not measured on ISS. Invasive measurement is out of reality, but now REG makes it possible to detect increased ICP and a cerebrovascular component of SANS with REG, non-invasively.

### Breath-holding

Breath-holding increases blood CO_2_ concentration, resulting in vasodilation in brain capillaries by increasing brain blood volume [52,53]. It is a clinical test of cerebrovascular reactivity (CVR) [59]. CVR is defined as the ability of vessels to alter their caliber in response to vasoactive factors, using dilation or constriction, to increase or decrease regional CBF. CVR may provide a sensitive biomarker for pathologies where vasculature is compromised. For the vasoactive stimulus, vasodilatory hypercapnia is usually induced through manipulating respiratory gases, including inhaling increased carbon dioxide concentrations. However, most of these methods require an additional apparatus and complex setups, which may not be well-tolerated by some populations and are also not widely available. For these reasons, strategies based on voluntary breathing fluctuations without the need for external gas challenges have been proposed [59].

### Head-down tilt (Trendelenburg) position

The ground-based models of microgravity are useful tools for determining the gravitational impact of spaceflight on the human body. The head-down tilt (HDT) bed rest, where the subject remains supine at −6 degrees for periods ranging from a few days to several weeks, is the most used ground-based model of microgravity for cardiovascular deconditioning. HDT can replicate cephalic fluid shift, immobilization, confinement, and inactivity [55,56].

In this descriptive study, our goals were: 1) to describe REG pulse wave morphology changes during/after breath-holding and in HDT position; 2) to calculate REGx.

## Materials and methods

### In vitro

#### Bioimpedance electrode

In clinical and research environments the REG and bioimpedance electrode can be a regular electrocardiogram (EKG) electrode. For military and space research a wearable, reusable, conductive fabric is a more real option. Previously we tested several conductive fabrics for this purpose [60]. For the latest test, we used a silver-covered plastic: Combat Medic Antimicrobial Silver Barrier Wound Care Dressing – Silverlon (https://www.silverlon.com/us). REG and circumferential arm bioimpedance electrodes were prepared and measured. The bioimpedance electrodes were made from Silverlon material. The size for REG was 3x4 cm. The measure of the arm electrodes was 22 and 26x4 cm. Both electrodes have a 4 mm diameter male metal snap button fastener (identical to EKG electrode contact). The resistance of the Silverlon was 1.5 ohm at a 10 cm distance, measured with a multimeter (Test Bench, BK Precision, Chicago, IL). The REG electrodes were placed above the eyebrows. The arm electrodes were placed on the lower arm at the wrist and the elbow fixed with a rubber holding band. There was no alcohol rubbing. A bipolar bioimpedance amplifier was part of the Cerberus system (Quintlab Ltd, Budapest, Hungary). The technical specifications of the impedance amplifier are: Six channels. Measuring current: 1 mA, 125 kHz, sinusoidal. Z0: max. 2 kohm. dZ: ±1 ohm. Bandwidth: 0.5 - 30 Hz. Noise: <2 mohm/50 ohm. The analog-digital converter was a USB-6211 (National Instruments, Austin, TX). The laptop was a G74S (ASUS, Taiwan, China). The recording program was a DataLyser (DL) with a 200 Hz sampling rate [61].

#### PR Pearsons's correlation coefficient (PRx/REGx)

**Fig. 1:**
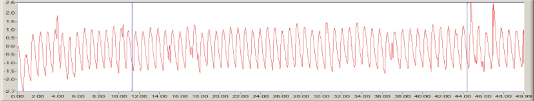
Trace of REG signal with Silverlon electrodes with Cerberus amplifier. The time window is 33.125 sec. The Y axis is in volt; the X axis is in second.

**Fig. 2:**
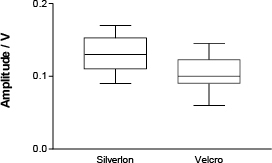
Boxplot of bioimpedance pulse peak values in Silverlon and Velcro electrodes (n=14 peaks). The difference is significant (p =0.0052).

Since the CBF AR status (PRx) calculation (Pearson’s correlation coefficient) [27] with REG is not common knowledge, we performed an in vitro demonstration. A function generator (BK Precision 3011B 2 MHz, Yorba Linda, CA) was used to imitate bioimpedance pulse waves on the head and arm. For analog-digital conversion, a USB-6211 (National Instruments, Austin, TX) was used. The recording laptop was a G74S (ASUS, Taiwan, China). For recording DataLyser (DL) [61] software was used with a 200 Hz sampling rate – Fig. 1 and Fig. 2. PRx/REGx calculation: The method of calculating secondary indices of CBF AR is based on the “moving correlation coefficient” [27]. This method allows analysis of the degree of correlation between two factors within a time series where the number of paired observations is large. Time-averaged values from each factor (10 seconds) were plotted in an x–y scattergram in a moving correlation window of 5 minutes and renewed every interval from 10 seconds to 1 minute. The correlation coefficients are calculated as a simple Pearson correlation coefficient and range from maximal −1 (negative correlation = CBF AR is active) to +1 (positive correlation= CBF AR is passive) and can be further analyzed as a time-dependent variable.

### In vivo

#### Subjects

We measured 19 healthy volunteers (6 females and 13 males). They were in a horizontal prone position on the tilting table. The length of the files was: 45.47 ± 5.45 (mean ± SD) minutes. The means for age (n=19) was 22.68 ± 1.49 years; height was 177.63 ± 6.18 cm, and body mass index was 22.94 ±2.43. There was no significant difference between the male and female groups. There was a significant group difference in weight (males 76.62 ± 7.16 kg and females 63.33 ± 3.98 kg; p=0.0001).

Abdominal circumference was measured at the navel level in a standing position. Abdominal circumference was significantly different (p=0.03): males (84.31 ± 5.41 cm) and females (75.17 ± 7.81 cm).

#### Preparation

Electrodes were placed while volunteers were in a sitting position. Before electrode placement, the skin was cleaned with benzine and EEG cleaning paste. The electrodes were regular electrocardiogram electrodes. The REG location was: bifrontal: Fp1-Fp2 and bitemporal: F7-F8 according to the EEG 10-20 International System of Electrode Placement [62]. Bioimpedance electrodes were placed on the left lower arm at the elbow and wrist, on hairless areas. During recordings, a face mask (Varifit, with AIR gel technology; Sleepnet Corporation, Hampton, New Hampshire) was used to measure exhaled CO_2_ concentration with a bedside monitor.

#### Equipment and Materials

A bedside monitor (BeneVision N15, Mindray North America, Mahwah, NJ) was used to record exhaled CO_2_ concentration and systemic arterial blood pressure (ABP). ABP was measured by the arm cuff on the left arm 6 times: 3 times during the control (horizontal supine position) period and 3 times during the HDT position, in both cases before 30-second breath-holding.

A bipolar bioimpedance amplifier (ReoRON-61, Medicor, Esztergom, Hungary) was used with an additional amplifier (BK-094-1; Elsoft BT, Budapest, Hungary) to amplify, filter, and switch symmetrical-to-asymmetrical signals. REG and lower arm bioimpedance signals were recorded together with the DL program.

The sampling rate of the analog signals was 200 Hz with DL software. The analog-digital converter was a USB-6211 (National Instruments, Austin, TX). Data collection was performed with a laptop (Alienware, Dell, Round Rock, TX). Event markers, as analog signals, were stored together with bioimpedance signals. Text notes were entered during the recording as events with time stamps which helped identify the challenges’ start and stop and numbers of actual ABP during data processing. DL creates deidentified files by automatically generating both waveform and note file names.

Challenges were as follows: 1) control/rest (0^o^) on tilting the table in the supine position for 20 minutes; 2) 30-sec breath-holding 3 times; 3) HDT (–15^o^) during about 20 min and 30-sec breath-holding 3 times. Between breath-holding there were 5 min rest.

#### Data processing

**Fig. 3:**
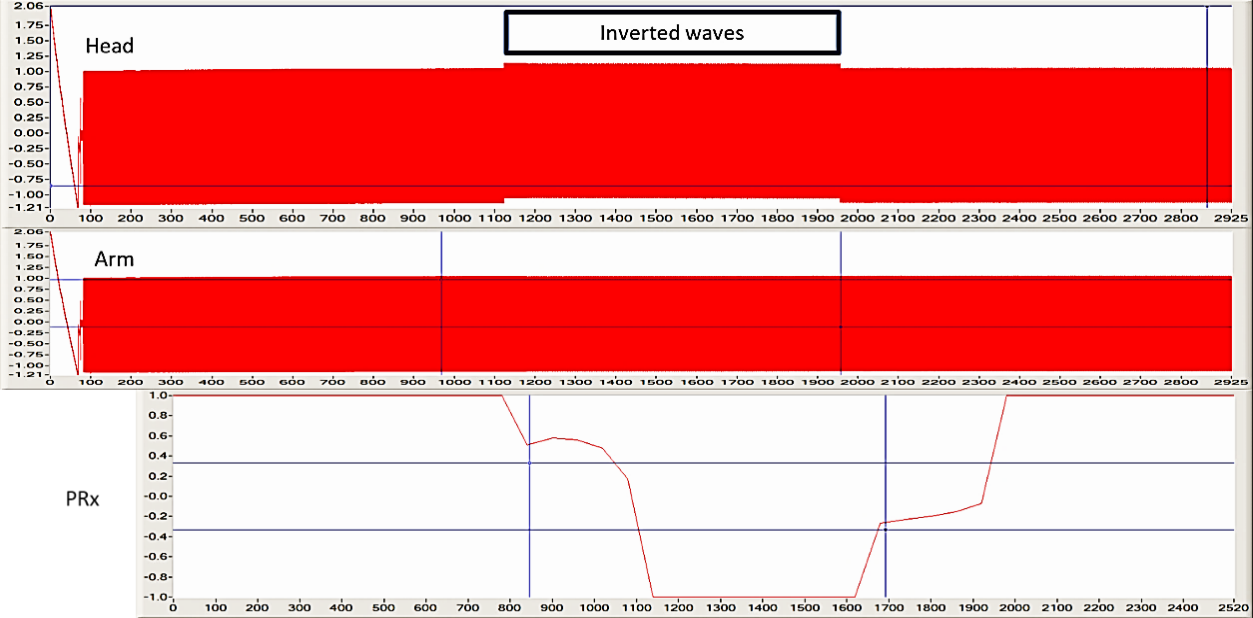
Upper two traces: 10 Hz sinus ±1 V labeled as head and arm, imitating REG, and arm bioimpedance pulses. Channels are in phase on the left and right sides; in the middle, the head pulses were inverted indicated by minimal amplitude increase—lower trace: Pearson’s correlation coefficient calculation (PRx). The left side indicates passive CBF AR, value is +1. The first PRx number is 300 sec. During the phase inversion, the value changes to −1, indicating active CBF AR status.

The DL software stores visualizes and processes analog physiological signals. The signals are stored in binary format. The metafile involves 1) setup information; 2) the time stamp and 3) entered text in ASCII format. The traces were exported as pictures in a PowerPoint (Microsoft, Redmond, WA) file, involving the notes and calculated variables such as REGx, Fig. 3. As a first step, 50 Hz interference contamination of the pulse wave was eliminated by smoothing with a 0.04-second window running average. Anacrotic time was measured by positioning cursors to the minimum and maximum values of the pulse waves. The amplitude difference was automatically calculated by the program and copied into an Excel spreadsheet (Microsoft, Redmond, WA). Eye blinking, talking, etc. artifacts containing waveforms were excluded from pulse wave reading. REG-derived variables were:

1. P1 and P2 amplitude of REG during control and HDT.
2. P1 anacrotic time: time from pulse wave minimum to maximum of P1.
3. Elapsed time from the start of 30-second breath-holding to maximal P1.
4. REGx calculation from bifrontal and bitemporal derivations + arm rheogram.

The REG pulse wave amplitude calculation was performed after visual inspection of the trace and selection of artifact-free pulses of 5-10-second lengths. During breath-holding, that period was selected which involved the maximum REG pulse amplitude. The DL program involves automatic peak detection. The number of maximum amplitudes was calculated with DL and copied into an Excel spreadsheet, and their differences to the control were calculated (t-test) for breath holding. REG peak delay was measured as the elapsed time in seconds between the manual marker indicating the command of the start of breath-holding to the time of maximal REG pulse amplitude. Additionally, the REG signal was analyzed with Pearson’s correlation coefficient [27] which is a DL menu and can operate in real-time as well.

For statistical analyses, we used Prism (GraphPad Software, Boston, MA) and Excel (Microsoft, Redmond, WA). Compared modalities were:

1. REG pulse wave P1 and P2 during control and HDT;
2. REG 1st peak (P1) amplitude during control/ baseline and 30-sec breath-holding or immediately after it;
3. REGx in bifrontal and bitemporal derivations;
4. Female-male groups. Results are presented as mean ± SD. P<0.05 was considered significant.

### Informed consent

Informed consent has been obtained from all individuals included in this study. After the information about the purpose and details of the tests, subjects signed the consent form.

### Ethical approval

The study was conducted in the Aeromedical, Military Screening, and Healthcare Institute, Medical Centre, Hungarian Defence Forces, Kecskemet, Hungary. The test procedure was conducted by the Declaration of Helsinki and the Ethical Board approved by the Institutional level at the Military Center of Hungarian Defence Forces on September 16, 2020.

## Results

### In vitro

#### Bioimpedance electrode

Sample REG signal recorded with Silverlon electrodes, Fig. 1. The arm bioimpedance sign was recorded with the Silverlon electrode and with conductive Velcro (Hi-Meg, Velcro, Manchester, NH) circumferential electrodes. Fourteen peaks were measured in both traces. The pulse amplitude mean (n=14) was higher than Silverlon: mean: 0.13; Velcro: 0.10. p=0.0052; 95% confidence interval: 0.009788 to 0.04521. See Fig. 2.

#### PRx/REGx

The phase inversion of the sine wave demonstrated by the PRx change from +1 (passive CBF AR) to –1 (active CBF AR), see Figures 1 and 2.

### In vivo

#### Systemic variables

The difference between horizontal/control and HDT position in systolic (p=0.47), diastolic (p=0.35) blood pressures, and heart rate (p=0.70) was not significant. The difference between female and male groups in systolic, diastolic pressures, and heart rate was not significant. The significant differences between the female and male groups were in weight (p=0.0001) and abdominal circumference (p=0.03). There was no significant difference in age (p=0.59); height (p=0.06); or body mass index (p=0.05). A typical recording is presented in Fig. 5.

**Fig. 4:**
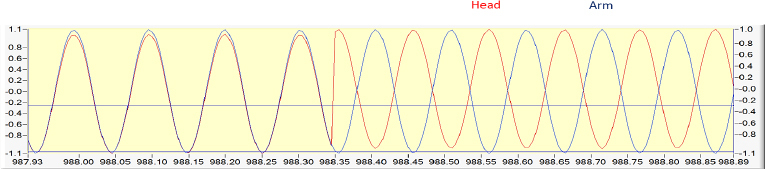
The two 10 Hz sinus ±1 V waves imitating head and arm pulse waves. On the left side in phase. On the right side is the inverted portion. The phase inversion starts at 988.35 seconds - time window: 0.965 min.

**Fig. 5:**
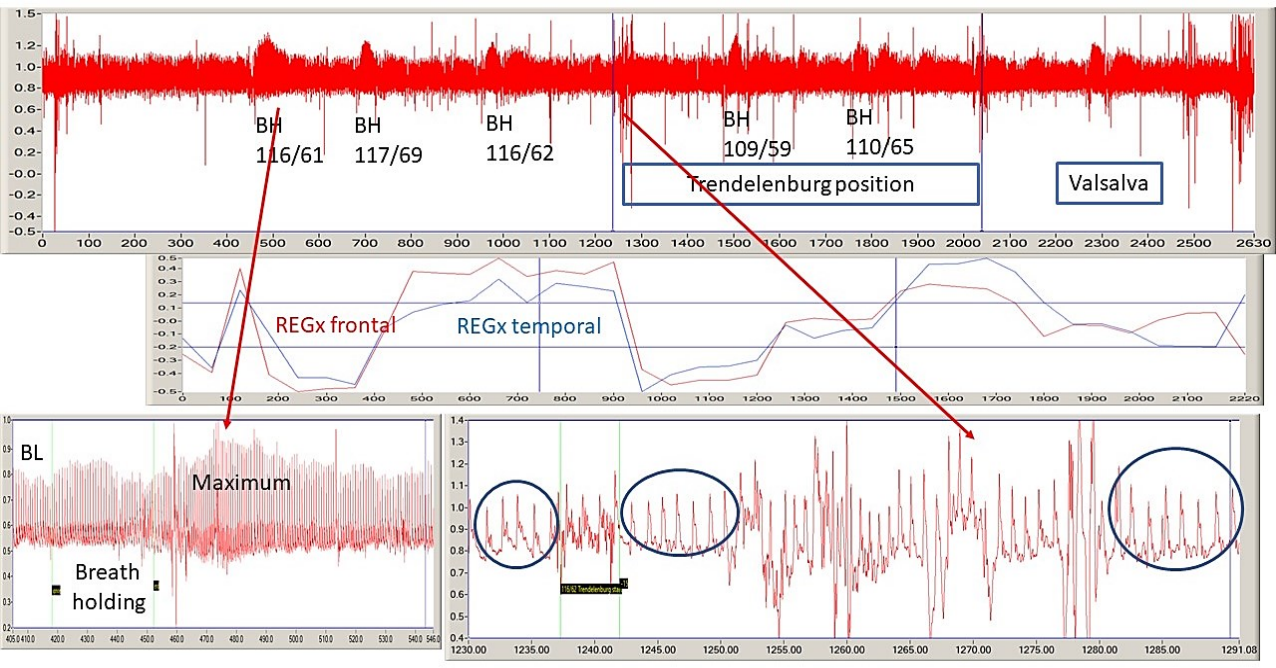
Trace of bifrontal REG (upper trace) on a tilting table. BH indicates the breath holdings. Under its label are the blood pressure (systolic/diastolic) numbers. The middle traces are the REGx traces. The lower trace shows fragments of REG during breath-holding (left side) and transition from horizontal to HDT position (right side). Arrows indicate the location of magnified portions. The Y-axis is in Volt. The X-axis is in seconds. The window size is 2630 seconds, 43.83 min. The file name is April 26 1.

#### REG pulse wave morphology during HDT position

**Fig. 6:**
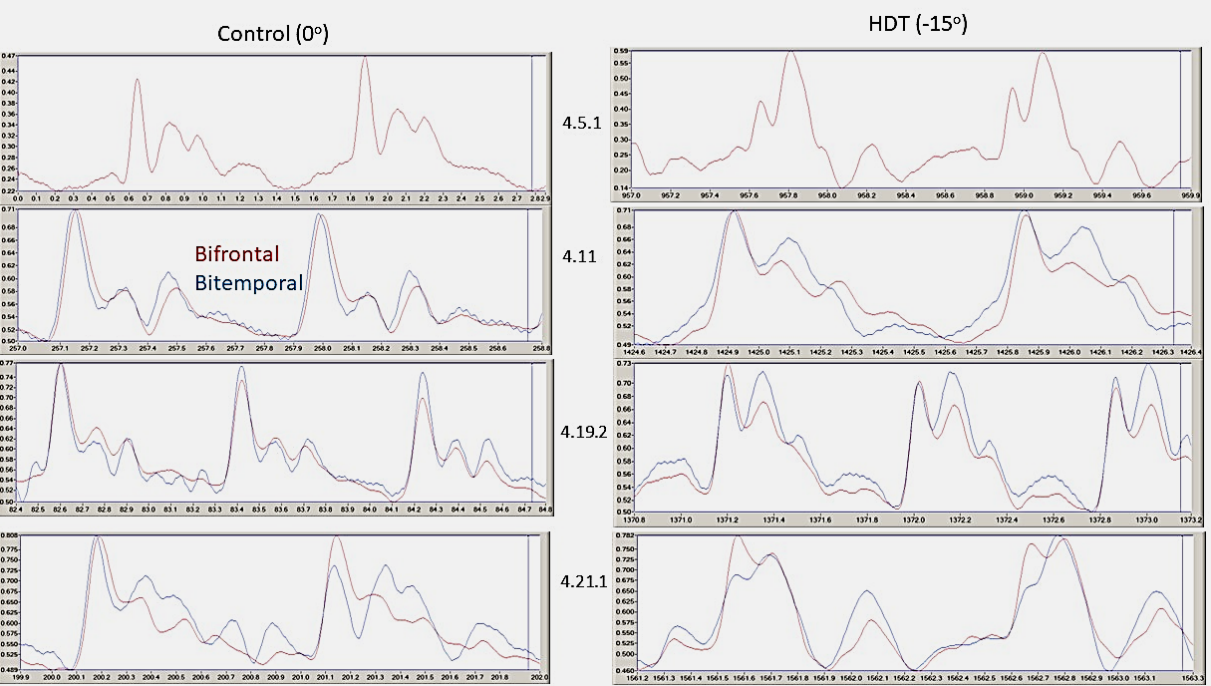
Fragments of recording of bifrontal and bitemporal REG pulse waves during control/rest (before HDT position; 0^o^), on the left side) and during HDT positions (-15^o^) (on the right side) in four subjects. The color indicates derivation. The Y axis is in Volt. X axis is in seconds. The window size is about 3 sec. Subjects are labeled by the recording date, for example, 4.5.1: the first recording that day.

During HDT position out of 19 subjects, only 10 (53 %) showed REG P1 amplitude increase (0.02 %; p=0.46). The increase or distortion of REG P2 during HDT position was typical in 17 cases; only 1 male and female did not show it. The difference of the P2 amplitudes between the control and HDT position for females was non-significant in only 1 case (out of 6 = 17 %) and for males was 5 (out of 13 = 38%) in bifrontal REG derivation. The 2^nd^ peak increased in 15 subjects (78 %); the “shoulder” formation on the catacrotic (descending) side was in 11 subjects (58%). The mean percentage increase was 6.94 % for females and 13.66 % for males. The increase was significant in 5 cases (out of 6; 83 %; P<0.0001; 95 % confidence interval –0.04123 to –0.02710) for females and 8 for males (out of 13; 62 %; P<0.0001; 95 % confidence interval –0.05796 to –0.05178). The P2 increase (compared to the control, i.e., before HDT) was significant only in the male group (p=0.003). The baseline REG P1 amplitude mean was 1.042 V (n=10) before breath-holding. Within seconds of the start of 30-second BH, there was a transient REG pulse amplitude increase. After the end of BH, the maximal REG 1 amplitude increased with a mean of 1.208 V (n=11). This increase is 15.93 percent BL. On the bottom of the right side is the magnified recording of the transition from rest/control to the HDT position. The change is that the 2^nd^ peak is increasing; see inside the circles. REG pulse wave comparison in control and during HDT is shown in Fig. 6. (Numerical values of these P2 increases are given in Table 1). Red and blue represent the bifrontal (red) and bitemporal (blue) REG derivations. The dominant morphological change is the P2 amplitude increase or “shoulder formation” on the decreasing, (catacrotic) side of the pulse wave during the HDT position. It is demonstrative and significant. P value P<0.0001; 95% confidence interval –0.04123 to –0.02710 for females and P<0.0001; 95% confidence interval –0.05796 to –0.05178 for the male group, see Fig. 5.

**Table 1:** REG P1 and P2 amplitudes (means of 5-10 pulse peak values) during control and HDT in four subjects, calculated from pulse waves presented in Fig. 6. The amplitude difference was calculated as a percentage of the control/baseline (BL%). REGx R2: correlation coefficient of bifrontal and bitemporal REGx during the full time of the recording. The sample traces are presented in Fig.6. Arm bioimpedance recordings were missing in the case of the 4.5.1 subject, consequently, there was no REGx calculation.

REG peak values and REGx correlation coefficients					
	P1	P2	P1	P2	REGx
April 5.1					R^2^
Control	0.43	0.35	0.47	0.37	N/A
HDT	0.42	0.59	0.47	0.58	
BL%	−0.33	36.32	26.27	57.97	
April 11					
Control	0.71	0.58	0.7	0.57	0.75
HDT	0.71	0.63	0.7	0.63	
BL%	−0.16	7.64	0.1	8.99	
April 19.2					
Control	0.77	0.67	0.73	0.67	0.65
HDT	0.73	0.73	0.7	0.67	
BL%	−4.74	8.91	−4.1	0.71	
April 21.1					
Control	0.81	0.66	0.81	0.67	0.55
HDT	0.78	0.74	0.76	0.77	
BL%	−2.89	12.01	−5.78	16.04	

#### REG pulse wave morphology during breath-holding

The REG traces show characteristic pulse amplitude increases caused by breath holdings during control and HDT positions (Fig. 6). The magnitude of these increases was not identical. Additionally, these increases have different maximums in time: some were within a few seconds after the start of breath-holding, some after their end, or both. REG P1 increased but it was not significant comparing control to HDT position for males (p=0.16) nor for females (p=0.53). The only significant differences were between male and female groups after the second (p=0.02) and the third 30-second breath-holding (p=0.01). There was no significant difference between male and female groups in REG P1 increases during the control period after 30-second breath-holding nor between control and HDT position.

There was no significant difference in mean REG P1 amplitudes during breath holding between 1) control (before HDT position) and during HDT position; neither 2) between male and female groups (male: 2.35 ± 7.5 % and female −0.27 ± 3.6 % (p=0.33).

**Fig. 7:**
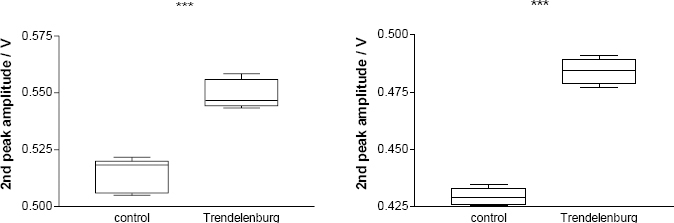
Change of P2 amplitude of REG pulse waves in female (n=6; on the left) and in the male group (n=13; on the right) before (control/baseline; 0o) and during HDT position (–15^o^). P<0.0001 in both groups. 95% confidence interval −0.04123 to −0.02710 in the female group and −0.05796 to −0.05178 for males.

#### REG P1 maximum time

REG P1 maximum time was measured during three 30-second breath-holding tests during the control period as well as during the HDT position. There was no significant difference between 1) control and HDT position (p=0.37): 2) males and females (P=0.79); 3) 1^st^, 2^nd^, and 3^rd^ apnea during control and HDT position. The range was from a few seconds to more than a minute. Also, in some cases, the elapsed time changed intra-individually.

#### CO_2_

There was no significant difference in the means of the end-tidal CO_2_ change before and after breath-holding between control (before HDT position) and during HDT position (control: 14.85 ± 6.81 %; and HDT position: 14.03 ± 6.76%; p=0.58). Also, there was no significant difference in end-tidal CO_2_ change between male and female groups in the control position (p=0.14) nor the HDT position (p=0.07). HDT did not influence the end-tidal CO_2_ concentration.

#### REG anacrotic time

REG anacrotic time was in the healthy range both for males (87.15 ± 10.54 msec) and females 86.03 ± 25.05 msec). The difference was not significant (P=0.43).

#### REGx

A typical recording and PRx graph are presented in Fig. 5. “Trendelenburg position” indicates its time: 13.36 minutes. Both REGx-es were turned active (trending to −1) after the first breath holding as well as during HDT position. Both male and female averaged REGx trace has a sharp decrease at the start of the HDT position, see Fig. 5. The male’s decrease was deeper than the female's. The difference between male and female REGx was significant (p=0.03); the correlation coefficient was 0.49. The average correlation coefficient between bifrontal and bitemporal derivations was 0.53 ± 0.31. There was no significant difference between the male and female group's bitemporal REGx average in bitemporal REGx (p=0.38) and the correlation coefficient was 1. During HDT position (between 15-37 minutes) both groups' REGx values decreased but the male values were more than the females (p=0.0002). This difference may be coincidental with the difference in abdominal circumference.

## Discussion

This observational study described REG morphological changes during 30-second breath-holding and in HDT position. This study is a continuation of previous work:

1. Early studies established a correlation between REG and other CBF measuring methods by using CBF AR tests [37–39].
2. A study clarified the physiological basis of the correlation between ICP and REG and PRx and REGx in an animal study by using the ICM+ program 29];
3. A study used neurocritical patients and presented a correlation between active/passive status of CBF AR and REG pulse wave morphological change [45].

### REG pulse wave morphology during HDT position

The novelty is that the REG pulse wave showed identical morphological alteration to the ICP pulse wave during ICP elevation/decreased intracranial compliance: the elevation of P2 during the HDT position. The cause of such change is the hampered venous outflow from the brain. This finding is important because a non-invasive method (REG) can be used to detect decreased intracranial compliance/ICP elevation instead of invasive ICP measurement.

**Fig. 8:**
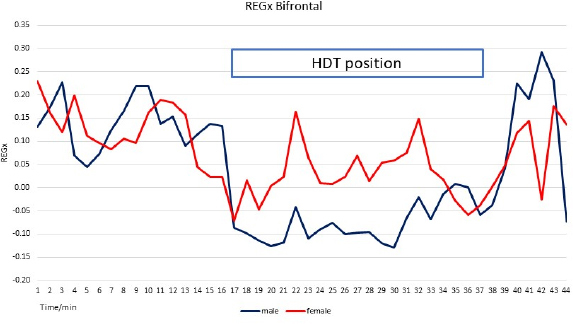
Averages of male and female groups bifrontal REGx during rest and HDT position. The correlation coefficient is 0.49; the statistical significance is 0.03. Note the difference during HDT. During this period, the correlation coefficient is 0.21; the statistical significance is p=0.0002. REGx decreased at the start of HDT, indicating the active status of CBF AR.

It was stated that ICP pulse pressure contains incredibly rich information on the cerebrospinal system dynamics, and therefore its analysis deserves wider recognition; “ICP is more than a number” [11]. Now we can apply this claim for REG as well, justified by our study. The background of these findings is that REG reflects cerebral volume and ICP increase as was described in previous publications [31,32].

### REG pulse wave morphology during breath-holding

Breath-holding is one of the CBF AR tests. Based on the previous conclusion, carbon dioxide is the most suitable vasoactive stimulus [52,53]. We used 30-second breath-holding to test CBF AR since it can be used on ISS as well. Details of central CO_2_ chemoreception can be found elsewhere [52]. The REG during and after breath-holding involved a faster and slower reaction as was described by [53]. There were individual differences in CO_2_ reaction seen in REG P1 increase and latency. If this test will be used on ISS or fighter pilots it is practical to record a control reaction first and compare later results to the control.

### CBF AR

**Fig. 9:**
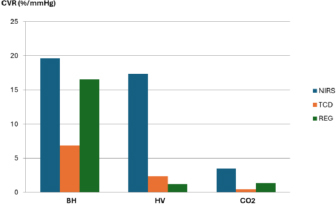
Comparison of cerebrovascular reactivity measured by 3 methods for 30 seconds breath-holding (BH), hyperventilation (HV), and CO_2_ inhalation (n=10). The figure was created from study data [41].

The different CBF AR mechanisms and their time scale were detailed by [24]. REG is unable to show any of here detailed CBF AR factors but rather reflects a summative picture referring to the arteriolar activity [63]. This is the source of many contradictory results: most clinical studies use transcranial Doppler (TCD) for the CBF measurement, which is not the arteriolar level of CBF i.e. reflects a different component of AR. Additionally, the myogenic mechanism acts on timescales faster than one second, the endothelial mechanism acts in one to two minutes, and the metabolic mechanism acts on different timescales depending on the circumstance. Still, it will not be reflected in dynamic CBF AR testing due to timescale constraints, and the neurologic mechanism acts within two seconds [18]. These facts can explain why we observed REG pulse amplitude increase a few seconds after the start of breath-holding and after the end of 30-second breath-holding as well. A human study [41] demonstrated that cerebrovascular reactivity number (%/mmHg) was higher for NIRS (19.65%) and REG (16.56%) than TCD (6.82%) during 30-second breath-holding – Fig. 9. This fact can be interpreted that both NIRS and REG reflect tissue and arteriolar AR while TCD shows the big vessel response (middle cerebral artery). A similar discrepancy was observed between REG anacrotic time and carotid flow change as a function of age, where the REG trend line slope was 10 times steeper than carotid flow [47].

### REGx

The guideline for the management of TBI from The Brain Trauma Foundation endorses the option to individualize management with ancillary monitoring, specifically citing the use of autoregulation monitoring and the pressure reactivity index (PRx) [64]. The PRx is a low-frequency linear correlation between mean arterial blood pressure (ABP) and ICP [65]. In health, the PRx is negative because intracranial blood volume (and therefore ICP) has slow fluctuations which are phase-shifted from similar slow fluctuations of ABP. When vascular reactivity is impaired, the PRx is positive because low-frequency intracranial blood volume changes are in phase with ABP changes [66]. Patients with cerebral perfusion pressure in a range that optimizes the PRx have better outcomes [66]. Real-time, invasive CBF AR monitoring is possible with the ICM+ program calculating pressure reactivity index (PRx). However, there is no standard for the normal oscillation of PRx. There will be no Institutional Review Board approving it. But with REGx it is possible to create a standard and related trigger, which is our goal.

A CBF AR-guided cerebral perfusion after traumatic brain injury demonstrated the use of targeting an individual and dynamic CBF AR-guided cerebral perfusion pressure is feasible and safe in TBI patients [68]. In that study, ICP was measured invasively. We started a study to compare invasive (PRx) and non-invasive (REGx) by using the ICM+ program in neurocritical care patients. The use of REG in neurocritical care may allow 1) the prevention of secondary brain injury; 2) the prediction of the prognosis of survival following head injury and optimization of CPP-guided therapy [68]. REG monitoring fits into the USU Surgical Critical Care Initiative (SC2i), which focuses on developing Clinical Decision Support Tools for critical care [69].

### Literature search (Aug. 13, 2023)

A PubMed search with keywords Trendelenburg position and REG pulse wave and Trendelenburg position and ICP pulse wave resulted in no hits. A PubMed search with the keywords head-down tilt and rheoencephalography resulted in 4 hits. Some used HDT –15 degrees, like we did. Another PubMed search with the keyword rheoencephalography resulted in 320 hits. Keywords with rheoencephalography and ICP resulted in 6 hits: 4 are ours. ICP and REG measurements were mentioned together in several publications [70–74]. A decrease in cerebrovascular reactivity was described in a rabbit study after antiorthostatis was measured by REG [73].

### Space medicine aspects

SANS microgravity-induced dysfunctions were detailed elsewhere [75–77]. A recent publication details the impact of spaceflight on CBF AR [56]. Systemic circulatory changes during space flight were described previously by using bioimpedance [76]. REG results were detailed by Kas'yan et al. Specifically, it was established that during flight under conditions of negative pressure on the lower half of the body, there was a decrease, in all cases, in pulse filling of brain blood vessels and an improvement in venous outflow from the cranial cavity [48]. Pulse filling means the decrease of the REG pulse wave’s first amplitude. REG pulse wave morphology change was not described in these studies. REG pulse wave analysis requires computerization and sampling rate above 100 Hz and a miniaturized REG device on the ISS. For space travel use of REG, conductive fabrics can be used as dry, reusable electrodes, without alcohol rubbing the skin, for example, the Combat Medic Antimicrobial Silver Barrier Wound Care Dressing (https://www.silverlon.com/us).

Use of REG

1. Helps measure the cerebrovascular aspect of SANS and ICP non-invasively.
2. Fits in NASA RFI Solicitation (Number: 80JSC020L0003 HRP) d. Cerebrovascular function.
3. Can help to create and test an adequate countermeasure for long-duration/deep space exploration.

Recommendations

1. Standardization of a CBF AR test (breath-holding) for increased CO_2_ concentration on ISS
2. Measure REG immediately before or after fundoscopy and optical coherence tomography scan on ISS.

This is a cross-disciplinary topic:

1. Such a device can also be modified as a life-sign monitor to record EEG, ECG, and respiration as well as to support dead-or-alive decision-making both in space and on Earth.
2. Other areas of research: Countermeasures. Since there was an inter-individual difference in the breath-holding reactions, the control/baseline number of the breath-holding test of an astronaut should be used to compare the result of the test on ISS and post-flight tests.

REG can determine the cerebrovascular aspect of SANS. By comparing previous REG use in space research, today we can generate new information about the status of intracranial compliance/ICP and CBF AR, such as REGx, which could not have been created decades ago. REG data has information on accelerated vascular aging during space travel since the lengthening of the REG anacrotic time reflects decreased compression chamber function and decreased elasticity of brain arteries [36,46,47]. REG use can quantify the effectiveness of applied counter-measures. REG measurement is suggested during the Artemis and Mars Exploration Program. Since there was no significant difference between bifrontal and bitemporal derivations, REG can be recorded from bifrontal electrode positions (above the eyebrows) better than bitemporal in the helmet for military use and in the headbands of astronauts. Microgravity during space travel (Moon and Mars) will have CBF/ICP/SANS actuality.

### Military aviation aspects

On board military fighter aircrafts, the possibility for human performance measurements, including mental and physical parameters is very limited. From the physiological (circulatory) aspect we have no information about actual fighting capability (compared to the detailed information of technical data in "black box" as flight recorder). From the flight safety aspect, it would be essential to understand and forecast just in time the pathophysiology of UPE (Unexplained Physiological Events/Incidents) which might lead to sudden incapacitation during flight even "on-mask" position.

Since 2002 the U.S. NAVY lost four F-18s, and the US Air Force one F-22 aircraft because the pilot was unaware of the sudden progression to an unconscious state flying at high altitude, being unable to switch on emergency oxygen and prevent fatal outcome [78]. During high G-s (accelerations and overloads due to the increased agility and maneuverability of combat aircraft) the inertial shift of blood to lower body parts can provoke blood pressure drop and pulse undulation leading to the transient cessation of brain perfusion and G-induced loss of consciousness (G-LOC). After military sorties longer deterioration of mental performance can commence due to A-LOC (almost LOC with changing brain perfusion during acceleration episodes) or hypoxia hangover (disturbed oxygen utilization for hours at brain cell level [79].

Continuous monitoring of biomedical data would be beneficial to prevent these scenarios, providing automatic feedback about circulatory parameters and tissue oxygen level (especially in the brain). From this aspect the CBF and NIRS alteration would be a valuable method, using dry electrodes inserted into the helmet, and possible warning signs are indicated on the pilot displays. From the technical aspect (during a real flight) the experience is still very limited. From the technical aspect (during a real flight) the experience is still very limited. Hopefully, soon, we can measure the perfusion changes in cerebral arteries, and we can evaluate the protective effect of Aircrew Equipment Assembly and breathing protocol [80,81]. Similarly, the follow-up for CBF changes would be essential to better understand the SANS / VIIP syndrome (referring to space-associated neuro-ocular symptoms including visual impairment and intracranial pressure increase driven by cephalad fluid shift and could easily threaten the success of deep space missions) [82].

Modern fighter planes are flying computers. They have no CBF problems but maybe software malfunctions. Unmanned combat plans are being tested these days. While there are pilots in these plans, we need to take care of them. One way to increase their safety is to monitor their CBF and its reaction to extreme gravitational stress. REG monitoring can serve this purpose since REG electrodes as conductive fabrics can be implanted into the helmet as well as the miniature amplifier. The good news is that the Canary^TM^ pilot's physiological monitoring system [80] is monitoring blood flow, heart rate, and SPO2 in their helmet (Elbit Systems Ltd. Haifa, Israel).

However, no study was published in which voice recording, brain electrical and circulatory parameters were compared to measure the sequence of cession of these signals. We made such a comparison in animal studies during lethal hemorrhage [83]. The loss of consciousness in fighter pilots during the rapid or gradual loss of cockpit hermitization and failure of the onboard oxygen generating system is caused by a transient decrease in cerebral oxygen utilization possibly accompanied by reduced CBF on a general or regional scale. A transient loss of consciousness in an aviator is illustrated by a current publication [84,85]. High G-forces cause blood flow to the brain to be impeded due to blood pooling low in the body under high acceleration. This causes the pilot to black out eventually. To prevent this, most if not all modern fighter pilots wear G-suits. The basic principle of operation is to constrict blood flow to the lower body to prevent pooling in a high G maneuver to increase blood flow to the brain. The G-suit typically buys the pilot about 1G of increased tolerance. The average human tolerance is between 3G and 5G. Good heart health can help mitigate the effects in sustained G situations. Therefore, fighter pilots must be in top physical shape as fighter planes are capable of very high G maneuvers. You can decrease the overall G-force experienced by altering your maneuver (i.e., not undergoing as much acceleration). However, in a combat situation, some high G maneuvers may be warranted and thus suits are used.

In civilian flying, all deliberate maneuvers are well within human limits. The prone position was extensively researched in Germany before anti-G suits were invented. The human body can tolerate G loads a lot better than sitting (86,87]. Modern, computer-controlled fighter jets can maneuver at a speed that the pilot can lose consciousness. An insidious phenomenon can occur when a pilot is exposed to +Gz stress even at insufficient levels to cause +Gz-induced loss of consciousness (G-LOC). Under these circumstances, the aircrew exhibited an altered state of awareness that was termed Almost Loss of Consciousness (A-LOC) by the U.S. Navy in the late 1980s. A-LOC is a syndrome that includes a wide variety of cognitive, physical, emotional, and physiological symptoms. While A-LOC has been observed in centrifuge studies and reported in flight for over 15 years, a definitive description of the syndrome does not exist. This study concluded that results may prove to be useful in designing closed-loop control systems for personal protective gear for pilots of high-performance aircraft [88]. The importance for fighter pilots is that cardiac output does not appear to affect the dynamic cerebral autoregulatory response to sudden hypotension in healthy controls, regardless of posture.

While fighter aircraft use pilots, a decrease in CBF will be a problem. It will be no problem in the future to use only unmanned combat aerial vehicles such as RQ-4 Global Hawk, X-47B (Northrop Grumman), or VISTA X-62A (Lockheed Martin) and MQ-25 Stingray Drone (Boeing) by using AI.

## Conclusions

1. The novelty of this work is documenting the first time that REG pulse amplitudes have an identical change to the ICP pulse wave morphology during decreased IC compliance/increased ICP during the HDT position. This is the physiological basis of the suggestion to use REG in neuromonitoring in ICU, military-medical use for transportation of wounded Service Members with traumatic brain/blast injury, hemorrhage, during hypotensive resuscitation, and space research as a non-invasive, surrogate measurement of ICP.
2. There is no standard for healthy CBF AR oscillation values since no IRB would approve such an invasive study. But with non-invasive REGx, it is now possible to create such a standard. Such a standard will help to create a REGx level of automatic trigger/alarm for staff interaction in the neurocritical care departments.
3. We initiated a correlative study by simultaneously using REG and invasive ICP and ABP in humans with the ICM+ program to compare invasive and non-invasive neuromonitoring as it was done in an animal study [29].
4. The previously questioned REG origin was documented as intracranial dominance [89].

### Limitations of the study

1. Described REG pulse wave alteration doesn’t offer mmHg number of ICP.
2. There is no standard for healthy people of PRx and REGx which would help to set up a threshold to trigger an alarm. Future studies can create a REGx standard for alarms.
3. There is no standard for the 30-second breath-holding test on Earth or ISS. Future studies can create these standards.
4. REGx calculation showed changes during HDT position, but we were unable to find correlative measured physiological modality yet. Future studies can clarify correlating physiological backgrounds.
5. The REG pulse waves involved artifacts of various origins which were able to avoid under visual inspection but not automatic deletion. Pattern recognition or artificial intelligence programs may solve this problem since artifacts can generate false results.
6. REG pulse wave was analyzed without artifacts only. In the future, artifact cleaning needs to be used.
